# Impact of the first national COVID-19 lockdown on referral of women experiencing domestic violence and abuse in England and Wales

**DOI:** 10.1186/s12889-022-12825-6

**Published:** 2022-03-15

**Authors:** Jasmina Panovska-Griffiths, Eszter Szilassy, Medina Johnson, Sharon Dixon, Anna De Simoni, Vari Wileman, Anna Dowrick, Elizabeth Emsley, Chris Griffiths, Estela Capelas Barbosa, Gene Feder

**Affiliations:** 1 The Big Data Institute, Nuffield Department of Medicine and The Queen’s College, University of Oxford, Oxford, UK; 2 Centre for Academic Primary Care, Population Health Sciences, Bristol Medical School, University of Bristol, Bristol, UK; 3 IRISi, Bristol, UK; 4 Nuffield Department of Primary Care Health Sciences, University of Oxford, Oxford, UK; 5 Wolfson Institute of Population Health, Barts and The London School of Medicine and Dentistry, Queen Mary University of London, London, UK; 6 School Arts and Social Sciences, City University of London, London, UK

**Keywords:** COVID-19 pandemic, Domestic violence and abuse, Interrupted-time series analysis, Non-linear regression, General practice, Primary care, Domestic violence and abuse referrals, School holidays

## Abstract

**Background:**

The lockdown periods to curb COVID-19 transmission have made it harder for survivors of domestic violence and abuse (DVA) to disclose abuse and access support services. Our study describes the impact of the first COVID-19 wave and the associated national lockdown in England and Wales on the referrals from general practice to the Identification and Referral to Improve Safety (IRIS) DVA programme. We compare this to the change in referrals in the same months in the previous year, during the school holidays in the 3 years preceding the pandemic and the period just after the first COVID-19 wave. School holiday periods were chosen as a comparator, since families, including the perpetrator, are together, affecting access to services.

**Methods:**

We used anonymised data on daily referrals received by the IRIS DVA service in 33 areas from general practices over the period April 2017–September 2020. Interrupted-time series and non-linear regression were used to quantify the impact of the first national lockdown in March–June 2020 comparing analogous months the year before, and the impact of school holidays (01/04/2017–30/09/2020) on number of referrals, reporting Incidence Rate Ratio (IRR), 95% confidence intervals and *p*-values.

**Results:**

The first national lockdown in 2020 led to reduced number of referrals to DVA services (27%, 95%CI = (21,34%)) compared to the period before and after, and 19% fewer referrals compared to the same period in the year before. A reduction in the number of referrals was also evident during the school holidays with the highest reduction in referrals during the winter 2019 pre-pandemic school holiday (44%, 95%CI = (32,54%)) followed by the effect from the summer of 2020 school holidays (20%, 95%CI = (10,30%)). There was also a smaller reduction (13–15%) in referrals during the longer summer holidays 2017–2019; and some reduction (5–16%) during the shorter spring holidays 2017–2019.

**Conclusions:**

We show that the COVID-19 lockdown in 2020 led to decline in referrals to DVA services. Our findings suggest an association between decline in referrals to DVA services for women experiencing DVA and prolonged periods of systemic closure proxied here by both the first COVID-19 national lockdown or school holidays. This highlights the need for future planning to provide adequate access and support for people experiencing DVA during future national lockdowns and during the school holidays.

**Supplementary Information:**

The online version contains supplementary material available at 10.1186/s12889-022-12825-6.

## Background

The COVID-19 infection which was first described in Wuhan, China in late 2019 became a pandemic that has profoundly affected all aspects of society worldwide and has been associated with complex changes in incidence and presentation of health problems [1]. To curb transmission of SARS-CoV-2, the virus that causes COVID-19, three periods of reduced social mixing (lockdown periods) were imposed throughout 2020 and 2021 in England and Wales [2]. These were associated with wide closures and changes of all aspects of society, including closing schools, a shift of clinical consultations in primary care from face-to-face to virtual (online) consultations and difficulties accessing primary care [3, 4]. These have made it harder for people to report complex health issues, including abuse [4, 5]. Remote consultations can produce barriers in accessing support where the perpetrator lives with the person seeking support; for example in disclosure of incidences and accessing support for Domestic Violence and Abuse (DVA).

DVA is a long-standing violation of human rights that damages health and wellbeing, globally experienced by about 1 in 3 women [6] and remains an ongoing social and health concern. It includes physical, psychological, sexual and financial abuse as well as coercive control and can be between current or former intimate partners or adult family members. Women experience more repeated incidents, greater abuse and longer impact than men [7]. It requires a multi-dimensional response with the health care services playing an important role in identifying women affected by DVA and referring them to DVA support programmes and services that can improve their safety and health outcomes [8]. In England and Wales, the majority of the support for people experiencing DVA comes from non-governmental organisations and charity sector DVA agencies [8]. However, general practice can provide a crucial link between people experiencing DVA and DVA support. The landmark national Identification and Referral to Improve Safety (IRIS) programme facilitated and monitored by IRISi, a social enterprise [9], has linked specialist DVA advocacy support with primary care [9]. It has been commissioned in 48 areas and has trained over 1000 practices (> 12%) in England, Wales, Northern Ireland and the Channel Islands, with over 20,500 women referred from these practices in the past 10 years. Existing work by our group has evaluated the impacts of implementing IRIS in a pragmatic randomised trial [10], followed by a post-trial ‘real world’ implementation study [11], showing that IRIS is effective and cost-effective [12], with a sustained impact, which lapses if funding is withdrawn [13], while a recent editorial highlighted the importance of support mechanisms for women experiencing DVA in the pandemic era [14].

Long periods where family groups are forced to spend time together, outside of the normal routine, can escalate and make it harder to disclose DVA [15, 16]. The severe physical distancing measures (‘lockdowns’) imposed by the United Kingdom (UK) Government from March 23, 2020 to control the spread of SARS-Cov-2 and its variants [2] are an example of enforced time together. These can have a detrimental impact on women experiencing DVA and their families [16–18]. For example, when the majority of DVA support mechanisms shifted to online during the lockdowns, services were less accessible to women experiencing DVA in their household. Isolation periods related to previous epidemics such as Severe Acute Respiratory Syndrome (SARS), have been associated with increased psychological distress, stress, depression, sleep disorders and problematic substance abuse [19]; these are all factors that may contribute to the triggering and escalation of violence [20, 21]. Evidence is starting to emerge from a number of countries that the COVID-19 pandemic and its lockdowns have led to an increase in DVA incidence [22, 23]. For example, France, Brazil and Italy have all reported an increase in domestic violence during 2020 compared to previous years [24]. In addition, emerging evidence from Refuge, the organisation running the 24-h national domestic violence helpline in England, suggests that calls to helplines have surged by 60% during 2020 compared to the equivalent period in the previous year [25]. These point towards evidence that the COVID-19 pandemic has resulted in an increase in DVA, but evidence on the impact of the COVID-19 national lockdowns on referrals from primary care to DVA support services is scarce.

Although the potential impact on DVA of societal lockdown – including loss of income and of contact with support networks - in response to a pandemic is likely to be more severe, there are some parallels between lockdowns and school holidays: added pressure to keep children occupied, and enforced family time spent outside of the normal daily routine. This could also lead to increased levels of abuse, and feelings of fear and isolation. The World Health Organisation (WHO) warned that longer time spent with abusive carers, without the safety of school services, will likely increase maltreatment and DVA incidence [26]. Calls to DVA helplines and police reporting of DVA have also been suggested to be lower during holidays and non-working days [27]. In England, emerging evidence also shows that fewer women are reporting domestic violence during the school holidays than outside of them [28]. Therefore, it is important to assess whether there is a school holiday effect on the number of referrals to services for women experiencing DVA and how comparable this effect is to that of the first national lockdown.

In this paper we present the results of the first analysis from the Primary Care Response to Domestic Violence and Abuse in the Covid-19 Pandemic (PRECODE) mixed study; the protocol for which has recently been published [29]. The aim of this paper is to evaluate the impact of large-scale systemic closures, in our case proxied by both national lockdowns or school closures, on the number of referrals of women from general practice to local IRIS DVA support programmes. To achieve this aim, we had two objectives. Firstly, we quantified the impact of the first national COVID-19 lockdown from 2020 on the number of referrals to DVA services in England and compare this to the same time period in the preceding year. Secondly, we evaluated the impact of the school holidays on the number of referrals to DVA services in the 3 years preceding the pandemic and the period just after the first COVID-19 wave.

## Methods

### Data

Across 33 IRIS commissioned sites, for which we had full datasets over the study period, we included data from women aged 16 and above, registered at each general practice between March 2017 to September 2020. We used anonymised data on daily referrals received by DVA specialists from general practices. We constructed a time-series of the data on daily number referrals using an indicator variable for the school holidays/lockdown period. We identified the optimal models that fitted the data (details in Additional file 1). For graphical display, we smoothed the observed average daily number of referrals using a 7-day moving average with uniform weights.

### Statistical analysis

The statistical analysis comprised interrupted-time series (ITS) and non-linear regression analysis on the daily time series of referrals to DVA services, similar to our previous work [13]. Two regression models were fitted to the data: negative-binomial model and Poisson model. For each regression model, we calculated the Akaike Information Criterion (AIC) and the Bayesian Information Criterion (BIC) to compare models, and chose the best-fit model based on the smallest values of these quantities (details are in Table S1 of Additional file 1).

To account for the period of the first national lockdown, the analogous time period in the preceding year and the periods of school holidays in 2017/2018, 2018/2019 and 2019/2020 school years, we added an indicator variable for days falling into these time periods listed in Table 1. This allowed us to estimate the difference in the number of referrals due to these events over the time period within which they occurred. Our analysis assumes that the effect from the lockdown and the school holidays on referral number is instantaneous, based on the understanding that circumstances where less social mixing is possible would immediately raise the barrier for General Practicioners (GPs) to make referrals for DVA.

**Table 1:** Timeline and reported mean number of referrals over different time periods that represent the Spring, Summer and Winter schools holidays in 2017–2019 and the period of the first national lockdown due to COVID-19 in 2020. These times are labelled in Fig. 1

Scenario (duration)	Start date	End date	Referral rate: mean [bias-corrected bootstrapped CI]		
			Over period before school holidays or lockdown	During period of school holidays or lockdown	Over period post school holidays or lockdown
Spring 2017 (12 days)	10.04.17	21.04.17	13 [3.48,22.52]	12.35 [11.52,13.22]	12.11 [11.14,13.08]
Summer 2017 (37 days)	26.07.17	01.09.17	11.23 [10.83,11.62]	9.7 [9.26,10.13]	11.4 [10.93,11.87]
Winter 2017 (17 days)	21.12.17	07.01.17	9.188 [9.637,9.743]	7.7 [6.723,8.676]	13.02 [11.926,14.078]
Spring 2018 (19 days)	29.03.18	16.04.18	11.58 [10.56,12.61]	9.33 [8,78,9.89]	10.5 [9.89,11.11]
Summer 2018 (48 days)	20.07.18	05.09.18	12.63 [11.92,13.35]	10.43 [0.46,11.82]	15.44 [14.75,16.14]
Winter 2018 (15 days)	19.12.18	03.01.19	14.62 [13.29,15.94]	12.3 [10.29,14.31]	12.25 [11.57,12.92]
Spring 2019 (15 days)	13.04.19	28.04.19	16.89 [15.54, 18.25]	14.14 [13.18 15.11]	13.1 [12.22,13.78]
Summer 2019 (48 days)	20.07.19	06.09.19	13.27 [12.67,13.87]	12.06 [11.45,12.67]	15.54 [14.62,16.47]
Winter 2019 (17 days)	20.12.19	06.01.20	16.36 [15.12,17.60]	9.27 [7.52,11.02]	18.05 [17.01,19.09]
March–June 2020 (1st national lockdown) (68 days)	23.03.20	01.06.20	16.98 [16.18,17.78]	11.25 [10.33,15.14]	14.23 [13.34,15.14]
March–June 2019 (analogous period to 1st lockdown) (68 days)	23.03.19	01.06.19	14.86 [14.24,15.48]	13.81 [13.18,14.44]	13.21 [12.79,13.62]
Summer 2020 (42 days)	20.07.20	01.09.20	15.54 [14.76,16.32]	10.28 [9.68,10.89]	11.18 [10.71,11.65]

For the best-fit model, we reported the incidence rate ratios (IRR) and 95% confidence intervals as indicators of change in referrals before and during the first national lockdown in 2020 and during the school holidays in the school year pre COVID-19.

## Results

The negative binomial model was the best-fit model for the data since both AIC and BIC were minimal for this model (see Table S1 in Additional file 1 for details). Table 1 shows the mean number of referrals to DVA services over all practices in the periods before, during and after the first national lockdown in 2020 and analogous time periods in 2019. Additionally, we also show the number of referrals before, during, and after each of the three spring, summer and winter school holidays for 2017–2019 and the summer holiday of 2020. Table 2 contains the IRRs and 95%CI values from the statistical analysis.

**Table 2:** Results from the statistical analysis showing the impact of the school holidays and the first national lockdown on the number of referrals to DVA services

Scenario	Incidence Rate Ratio [95% CI]	Bootstrap Standard error	*p*-value
Spring 2017	0.929 [0.691,1.25]	0.141	0.630
Summer 2017	0.865 [0.755,0.994]	0.059	0.036
Winter 2017	0.811 [0.561,0.902]	0.087	0.005
Spring 2018	0.837 [0.690,1.017]	0.083	0.073
Summer 2018	0.871 [0.789,0.962]	0.044	0.007
Winter 2018	0.956 [0.791,1.156]	0.092	0.646
Spring 2019	0.948 [0.827, 1.087]	0.066	0.447
Summer 2019	0.849 [0.771, 0.937]	0.043	0.001
Winter 2019	0.557 [0.457, 0.680]	0.056	*p* < 0.001
March–June 2020 (1st lockdown)	0.727 [0.661, 0.787]	0.032	*p* < 0.001
March–June 2019 (analogous period to 1st lockdown)	0.984 [0.905, 1.068]	0.042	0.707
Summer 2020	0.797 [0.707,0.898]	0.048	*p* < 0.001

Overall, the number of referrals to DVA services was 19% lower during the first national lockdown compared to the same period the year before (Table 1). During the first national lockdown in 2020, the number of referrals to DVA services declined significantly (27%, 95%CI = (21,34%), *p* < 0.001) compared to the period before and after. Over the same period of the lockdown the year before (March–June 2019), the change in referrals was less and not significant (2%, 95%CI = (− 7,10%), *p* = 0.707) compared to before and after (Table 2).

The number of referrals also declined during the school holidays, compared to before and after. During the short (~ 2 weeks) spring holidays, there was a small reduction (5–16%) in the number of referrals to specialist DVA services: 7% (95%CI = (− 25,30%)) in 2017, 16% (95%CI = (− 2,30%)) in 2018 and 5% (95%CI = (− 8,17%)) in 2019 (Table 2). During the long (~ 6 weeks) summer holidays there was a substantial reduction in referrals to specialist DVA services: 13% (95%CI = (3,25%)) in 2017, 13% (95%CI = (4,21%)) in 2018, 15% (95%CI = (6,23%)) in 2019 and 20% (95%CI = (10,29%)) in 2020. During the winter holidays 2017–2019 (which varied in length between 2 and 3 weeks duration), in 2019 there was a large (44%, 95%CI = (32,55%) reduction in referrals, while in 2017 and 2018 the reduction was less (18.9%, 95%CI = (10,31%) and 4.4%, 95%CI = (− 15,20%)).

**Fig. 1:**
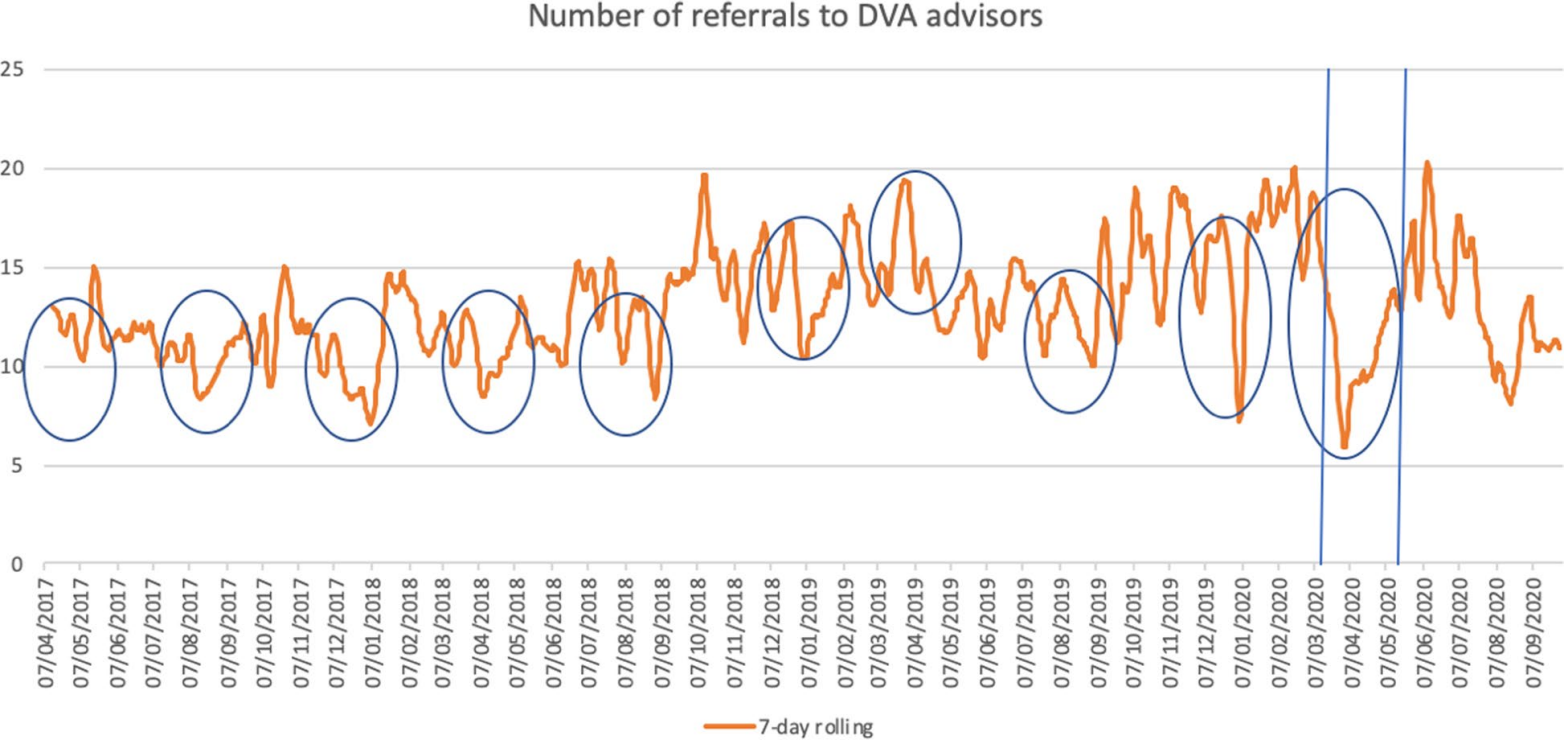
Timeseries of referrals to DVA services as a 7-day rolling average over the period April 2017 to September 2020. The solid vertical lines represent the start and end of the first national lockdown due to COVID-19 transmission in 2020. We observe a large decline in the number of referrals during the first national lockdown and a slower increase after the relaxing of the lockdown measures. There is also an observed decline in referrals during the schools holidays in 2019, but this decline is smaller than during the first national lockdown

**Fig. 2:**
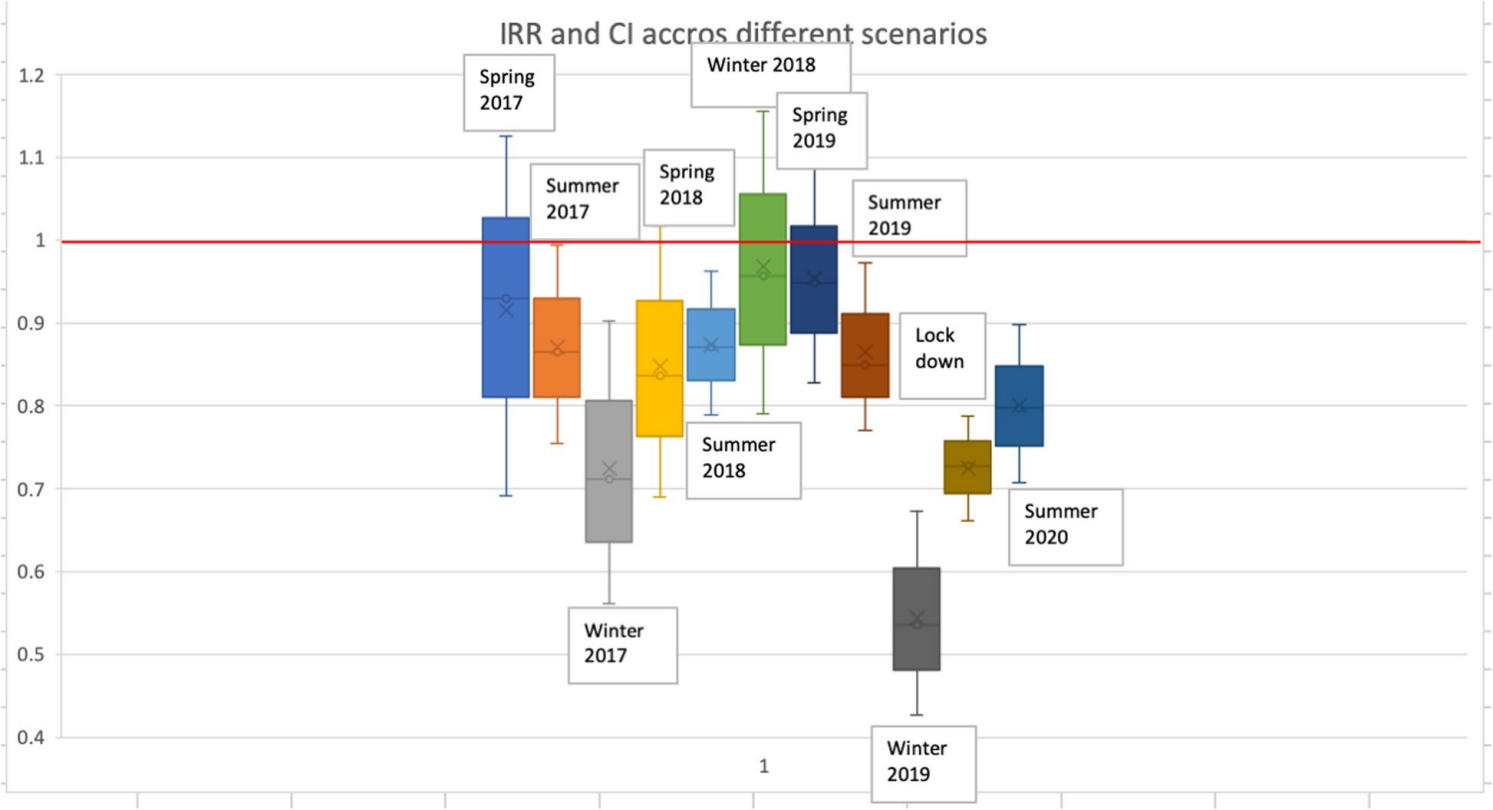
Box-plot of the impact of the schools holidays and the 1st National lockdown. The plot shows the Incidence Rate Ratios (IRR), as mean value, and the 95% Confidence Intervals, as whiskers, across the different scenarios. The decline in the number of referrals corresponds to IRR < 1 i.e. below the red horizontal line on the plot

Figure 1 shows the corresponding 7-day rolling average smoothed time-series, and we clearly see a steep decline in the number of referrals during the first national lockdown and some of the school holidays. The highest reduction in referrals was evident during the winter 2019 pre-pandemic school holidays (44%, 95%CI = (32,55%)) followed by the effect from the first national lockdown (27%, 95%CI = (21,34%)) and of the summer of 2020 school holidays (20%, 95%CI = (10,29%)) (Fig. 2).

## Discussion

This paper presents the results of the first analysis from PRECODE (Primary Care Response to Domestic Violence and Abuse in the Covid-19 Pandemic) mixed-methods study [29]. We quantified the number of referrals of women from general practice to the IRIS DVA support service during large-scale systemic closures in England and Wales, proxied by the first national lockdown in 2020 and the school holiday closures 2017–2020. Our findings show that the first national lockdown in 2020, between March and June 2020, led to a 27% reduction of referrals to DVA services compared to the period before or after the lockdown and 19% less compared to the same period the year before. A reduction in the number of referrals was also evident during the school holidays: with 44% fewer referrals during winter 2019 and 20% fewer referrals during the summer 2020 school holidays, followed by a smaller reduction (13–15%) in referrals during the longer summer holidays 2017–2019; and some reduction (5–16%) during the shorter spring holidays 2017–2019.

Our findings are evidence that during periods of systemic closure such as during the first COVID-19 national lockdown in 2020 and during the school holidays 2017–2020, there was a decline in the number of referrals to a primary care linked DVA support programme. This reduction in referrals contrasts the reported increases in domestic violence reports e.g. 30% increase in reports of domestic violence in France and 40–50% in Brazil during 2020 [24]. Similarly, the calls to the 24-h national domestic violence helpline managed by Refuge in England have been estimated to have surged by 60% during 2020 compared to equivalent period in the previous year [25]. Our findings are in line with the previously highlighted ‘pandemic paradox’ [30]: a disconnect between the increased incidence and severity in DVA over the pandemic period (and wider enforced closure periods) and the reduction in referrals to specialist DVA services. This disconnect suggests that during systemic closures such as the COVID-19 lockdowns, it is more difficult to disclose DVA within primary care health settings, although contact with DVA helplines increased [31]. One potential reason for this is that it may be harder for women to seek help and disclose violence or abuse when perpetrators are likely to be present such as during enforced systemic closures which in this study are proxied by the first COVID-19 lockdown and school holiday periods.

Our study is the first to evaluate the impact of the pandemic and the associated first national lockdown and compare it to other systemic closures, such as school holidays when the family is forced to spend prolonged periods of time together. Our findings of reduced number of referrals to a specialist DVA programme over these periods, combined with reported increase in the number of calls to domestic violence helplines [25, 32], points towards a negative association between ability to seek advice and help for women experiencing DVA and prolonged periods of system closures. Hence, it is crucial to develop alternative avenues of support during closure periods that may be more accessible and to explore how support can be resourced in the context of a widely impacted health system in the case of future epidemic waves. We need research, co-produced with DVA survivors and agencies to develop and evaluate appropriate support for people experiencing DVA during both potential future national lockdowns and during the school holidays.

Liaising closely with the National Domestic Abuse helpline Refuge (https://www.refuge .org.uk) our further research will explore the relationship between calls to helplines and referrals to primary care-linked DVA support programmes over a longer time period that includes the first as well as the subsequent COVID-19 epidemic waves in England.

The analysis in this study follows our previous multi-disciplinary research that utilises a rich data set of DVA referrals from a large number of practices which is statistically analysed with interrupted-time-series and non-linear regression analyses. This modelling approach is widely accepted as a robust and efficient method for evaluation of public health and primary care evaluations [27, 28]. In particular, regression modelling is useful in drawing conclusion for the duration of the study, and when considering specific time points of interest. A possible alternative would be to develop and utilise dynamic temporal models that use the data to calibrate mechanistic models for DVA analogous to infectious disease spread and calibrate to the historic pattern, which could be used to make future prediction; but this was beyond the scope for this study.

This work has some limitations. First, the data used in this analysis are from general practices trained by the IRIS programme which are part of an established referral route, but available to less than 20% of practices nationally. Further work is needed to generalise this to the wider UK and international context. Second, in this work we have not compared the police reports on how the pandemic has affected the DVA reporting. Reports from the Office of National Statistics (ONS) suggest that the police recorded 7% increase in offences flagged as domestic abuse-related in the period March to June 2020 compared to the same period in 2019 and 18% increase from the same period in 2018 [30, 31]. Additionally, reports from specialist services in South Wales suggest that there was little increase in the overall volume of police referrals during the lockdown, but the proportion assessed as high risk increased notably [33]. Within these reports the data is aggregated numbers and not a time series, and future work will look to obtain this data as time series and analyse it alongside the time-series of calls to the DVA helpline and the number of referrals to the IRIS programme. Understanding how these three measures correlate with each other, before, during and after the three pandemic waves, will allow for a more detailed analysis on the impact of the COVID-19 pandemic on DVA in England.

Another limitation of our study is that we have simplified the differences between national lockdowns and school closures as periods of systemic closure. The motivation behind using school holiday periods as a comparator to national lockdowns was the understanding that children are at home during the holidays, making accessing services more difficult. This, to some extent, might be comparable to how enforced time spent together in the lockdown, and possibly with the perpetrator present more, makes services less accessible. While this simplifying assumption was analytically adequate for this study, we note significant differences between school holidays and national lockdowns that our study has not addressed. For example, during the lockdown many children were attending online lessons and many parents were working from home, creating a more pressurised environment than those of school holidays. Additionally, the risk of COVID-19 infection acted as an additional barrier to help-seeking and service access. These important differences between the “stay at home order” during the first national lockdown and school holidays were not captured in our study.

Overall, this paper presents the first analysis of a mixed-methods PRECODE study [32]. As further data on DVA referrals in IRIS implementation settings become available for late 2020 and 2021, we will explore the determinants of risk of increased DVA incidences during different COVID-19 waves and the longer-term impact of COVID-19 on DVA in England.

## Conclusion

In summary, our study suggests that systemic closure periods proxied here by the first national lockdown in 2020 and the school holidays 2017–2020 led to declined number of referrals to DVA services in England, despite reported increased DVA incidences. This highlights the need to provide adequate access and support for people experiencing DVA during both potential future national lockdowns to suppress COVID-19 spread and during the school holidays.

## Supplementary Information

**Additional file 1. **Details of the statistical analysis. Time-series of the number of referrals to DVA advisors in England and Wales and regression models used in the analysis.

## Data Availability

The datasets used and analysed during this study and the numerical codes used to generate the outcomes of this paper are available from the corresponding author on reasonable request. Access will be subject to a data access agreement and following approval from the Chief Investigator and the University of Bristol Data Access Committee.

## Abbreviations

DVA: Domestic violence and abuse
IRIS: Identification and Referral to Improve Safety
GP: General practitioner
NHS: National Health Service
IRR: Incidence rate ratio
ITS: Interrupted time series
CI: Confidence interval
