# Supplementary material for: Impact of the first national COVID-19 lockdown on referral of women experiencing domestic violence and abuse in England and Wales
Source: BMC Public Health. 2022 Mar 15;22:504. doi: 10.1186/s12889-022-12825-6 (PMC8922060; doi:10.1186/s12889-022-12825-6)
Supplement: Supplementary file 1 — Additional file 1. Details of the statistical analysis. Time-series of the number of referrals to DVA advisors in England and Wales and regression models used in the analysis. [file 12889_2022_12825_MOESM1_ESM.pdf]

Appendix A
Details of the statistical analysis

Jasmina Panovska-Griffiths[1,2*], Eszter Szilassy[3], Medina Johnson[4], Sharon Dixon[6], Anna De Simoni[5], Vari Wileman[5], Anna Dowrick[6] , Elizabeth Emsley[3], Chris Griffiths[5], Estela Capelas Barbosa[3,7], Gene Feder[3]

[1]The Big Data Institute, Nuffield Department of Medicine

[2]The Queen's College, University of Oxford, Oxford, UK

[3]Centre for Academic Primary Care, Population Health Sciences, Bristol Medical School, University of Bristol, Bristol, UK

[4] IRISi, Bristol, UK

[5]Wolfson Institute of Population Health, Barts and The London School of Medicine and Dentistry, Queen Mary University of London, London, UK

[6] Nuffield Department of Primary Care Health Sciences, University of Oxford, Oxford, UK

[7] School Arts and Social Sciences, City University of London, London, UK.

*Corresponding author: jasmina.panovska-griffiths@ndph.ox.ac.uk

Overview

This appendix gives the details of the statistical analysis on the anonymised data from daily referrals received by DVA service providers across 33 IRIS commissioned sites, including data from female patients aged 16 and above, registered at each general practice between March 2017 to September 2020.

We present the results of the non-linear regression analysis on the daily referrals time-series using two regression models: negative-binomial model and Poisson model. For each regression model, in Table S1 we show the Akaike Information Criterion (AIC) and the Bayesian Information Criterion (BIC) to compare models, showing how the best-fit model was chosen based on the smallest values of these quantities.

Methods

The data was coded using STATA software (version 15.1). The analysis comprised the following steps:

1. Using time and a disruption predictor variable, we compared a negative binomial model and Poisson model projecting the corresponding Akaike Information Criterion (AIC) and Bayesian Information Criterion (BIC) values. Based on these values we chose the best-fit model for the analysis.

2.Time series was constructed from the number of daily referrals across all IRIS practices. We fitted the best-fit model to the data. To account for the period of the first national lockdown, the analogous time period in the preceding year and the periods of school holidays in 2017/2018, 2018/2019 and 2019/2020 school years, we used an indicator variable for days falling into these time periods listed in Table 1 of the main paper.

3. For the best-fit model, we projected outcome variables: incidence rate ratios (IRRs) with 95% CI and the p-value for the lockdown/school holidays indicator variable.

Results

1. Comparing different regression models

The calculated AIC and BIC values for a negative binomial model and a Poisson model as presented in Table S1. Based on the smallest AIC and BIC values, the negative binomial regression model was a better fit model.

Scenario	Negative binomial model		Zero-inflated Poisson model	
	AIC	BIC	AIC	BIC
Spring 2017	144.68	149.08	148.23	153.12
Summer 2017	404.13	411.66	406.12	413.45
Winter 2017	90.83	93.66	92.12	95.34
Spring 2018	206.79	212.28	209.23	215.12
Summer 2018	646.84	655.49	647.23	657.45
Winter 2018	194.00	198.99	195.34	200.12
Spring 2019	313.04	319.47	315.45	323.34
Summer 2019	660.44	669.16	664.23	671.23
Winter 2019	244.90	252.30	190.00	194.84
March-June 2020 (1st lockdown)	985.93	995.62	987.93	1000.85
March-June 2019 (analogous period to 1st lockdown)	879.19	888.77	882.34	912.77
Summer 2020	508.19	516.214	518.23	526.26

Table S1: Results from comparing different regression models that fit the data

2. For the referrals time series, we identified the best-fit model to be

$$log(\hat{Y}_{ij}) = (\beta_0 + u_{0j}) + \beta_1(time_{ij}) + \gamma PAUSE_{ij} + log\left({PracticeSize_{ij}}/{10000}\right), u_{0j} \sim N(0, \sigma_u^2) \quad (1)$$

where $\hat{Y}_{ij}$ is the estimated number of referrals for practice j at time i, assumed to follow a negative binomial distribution, u_{0j} is a random intercept term, which varies between practices, $PAUSE_{ij}$ is an indicator variable coded 0 for days at which there was no lockdown or school holidays, and 1 for days during the lockdown and school holidays. Different models were fitted to each scenario concerning the first national COVID-19 lockdown and the school holidays 2017-2019 as shown in Table S2.

Scenario	β_0	β_1	γ
Spring 2017	2.6417	-0.0071	-0.0729
Summer 2017	2.4321	-0.0001	-0.1451
Winter 2017	-0.3899	0.0102	-0.2945

**Spring 2018**	2.7011	-0.00082	-0.1771
**Summer 2018**	1.7360	0.00183	-0.1373
**Winter 2018**	5.6390	-0.0048	-0.0445
**Spring 2019**	5.5309	-0.00385	-0.0531
**Summer 2019**	1.2539	0.00164	-0.1625
**Winter 2019**	0.3703	0.00248	-0.5836
**March-June 2020 (1st lockdown)**	4.105	-0.00121	-0.3283
**March-June 2019 (analogous period to 1st lockdown)**	3.2842	-0.0009	-0.0160
**Summer 2020**	7.1662	-0.0038	-0.2263

Table S2: Coefficients of the regression models.

3. Using the negative binomial model, with predictor variables for time and the period of the lockdown and school holidays, the IRRs and their 95% CI and the corresponding p-values for the disruption predictor variables are given in Table S3 and also Table 2 of the main paper.

Scenario	IRR [95% CI]	Bootstrap Standard error	p-value
**Spring 2017**	0.929 [0.691,1.25]	0.141	0.630
**Summer 2017**	0.865 [0.755,0.994]	0.059	0.036
**Winter 2017**	0.811 [0.561,0.902]	0.087	0.005
**Spring 2018**	0.837 [0.690,1.017]	0.083	0.073
**Summer 2018**	0.871 [0.789,0.962]	0.044	0.007
**Winter 2018**	0.956 [0.791,1.156]	0.092	0.646
**Spring 2019**	0.948 [0.827, 1.087]	0.066	0.447
**Summer 2019**	0.849 [0.771, 0.937]	0.043	0.001
**Winter 2019**	0.557 [0.457, 0.680]	0.056	p<0.001
**March-June 2020 (1st lockdown)**	0.727 [0.661, 0.787]	0.032	p<0.001
**March-June 2019 (analogous period to 1st lockdown)**	0.984 [0.905, 1.068]	0.042	0.707
**Summer 2020**	0.797 [0.707,0.898]	0.048	p<0.001

Table S3: Projections from the mixed-effects negative binomial model from equation (1) for each borough.
